# The associations between oxidative stress and epilepsy: a bidirectional two-sample Mendelian randomization study

**DOI:** 10.1186/s42494-024-00173-4

**Published:** 2024-12-01

**Authors:** Lan Zhang, Ningning Zhang, Xuyan Sun, Sirui Chen, Yuanhang Xu, Yaqing Liu, Junqiang Li, Dadong Luo, Xin Tian, Tiancheng Wang

**Affiliations:** 1 The Second Hospital & Clinical Medical School, Lanzhou University, Lanzhou, 730030 China; 2 Department of Neurology, Epilepsy Center, The Second Hospital of Lanzhou University, Lanzhou, 730030 China; 3 Department of Neurology, The First Affiliated Hospital of Chongqing Medical University, Chongqing Key Laboratory of Neurology, Chongqing, 400016 China; 4 Key Laboratory of Major Brain Disease and Aging Research (Ministry of Education), Chongqing Medical University, Chongqing, 400016 China

**Keywords:** Oxidative stress, Epilepsy, Mendelian randomization, Causality, Serum metabolites

## Abstract

**Background:**

Studies on the association between oxidative stress and epilepsy have yielded varied results. In this study, we aimed to investigate the causal relationship between oxidative stress markers and epilepsy.

**Methods:**

A bidirectional two-sample Mendelian randomization (MR) study was performed based on publicly available statistics from genome-wide association studies. To explore the causal effects, single nucleotide polymorphisms were selected as instrumental variables. Inverse-variance weighted method was performed for primary analysis, supplemented by weighted median, MR-Egger, simple mode, and weighted mode. Furthermore, sensitivity analyses were performed to detect heterogeneity and pleiotropy.

**Results:**

Our results showed that part of the oxidative stress biomarkers are associated with epilepsy and its subtypes. Zinc is associated with increased risk of epilepsy and generalized epilepsy (odds ratio [OR] = 1.064 and 1.125, respectively). Glutathione transferase is associated with increased risk of generalized epilepsy (OR = 1.055), while albumin is associated with decreased risk of generalized epilepsy (OR = 0.723). Inverse MR analysis revealed that epilepsy is associated with increased levels of uric acid and total bilirubin (beta = 1.266 and 0.081, respectively), as well as decreased zinc level (beta =  − 0.278). Furthermore, generalized epilepsy is associated with decreased ascorbate and retinol levels (beta =  − 0.029 and − 0.038, respectively).

**Conclusions:**

Our study presented novel evidence of potential causal relationships between oxidative stress and epilepsy, suggesting potential therapeutic targets for epilepsy.

**Supplementary Information:**

The online version contains supplementary material available at 10.1186/s42494-024-00173-4.

## Background

Epilepsy is one of the chronic neurological disorders that has affected more than 70 million people worldwide [1, 2]. It has profound impacts on individual health, family burden and social economy. Despite numerous research, the etiology and pathogenesis of epilepsy remain to be further explored. Generally, the development of epilepsy is a result of imbalance between excitatory and inhibitory neurotransmitters. Multiple factors can cause inhibition of inhibitory neurons or overactivation of excitatory neurons, causing abnormal electrical activity and eventually leading to seizures. The causes of epilepsy mainly include genetics, inflammation, trauma and some other unknown factors [3]. Oxidative stress is increasingly recognized to play an important role in the physiological process of epilepsy.

Previous studies have shown that patients with chronic epilepsy have a significantly increased level of oxidative stress compared with non-epilepsy individuals [4]. By evaluating the levels of antioxidant enzymes in the resected hippocampi of nine patients with temporal lobe epilepsy and hippocampal sclerosis [5], researchers found significantly higher levels of antioxidant enzymes in the sclerotic hippocampi compared with the control group. Glucose transporter type 1 (GLUT1) deficiency syndrome is characterized by seizures in early infancy. Studies have found that the modified Atkins diet can decrease the cerebrospinal fluid levels of oxidative stress markers in GLUT1-deficiency patients [6]. This diet has become a very effective treatment method to reduce such a seizure [7]. Besides, increased levels of reactive oxygen species (ROS) and oxidative stress have been observed in a mouse model of infection-induced temporal lobe epilepsy [8]. A study analyzing antioxidant enzyme activities in a genetic epilepsy susceptible rat model revealed a lower glutathione (GSH)/glutathione oxidized ratio compared to control Sprague–Dawley rats, indicating heightened oxidative stress [9]. Further experimental studies showed that the absence of expression of antioxidant enzymes, such as superoxide dismutase (SOD), leads to increased mitochondrial oxidative stress [10], thereby increasing the risk of epilepsy. These findings provide insights into the potential role of oxidative stress in epilepsy.

A balanced oxidative stress status is known to play a crucial role in the normal and orderly functioning of the body [11]. Oxidation of proteins may lead to changes in the physiological functions of proteins. Excessive levels of peroxides lead to oxidation and chemical modification of biological macro-molecules, thereby participating in the pathological mechanisms of different diseases. Due to the high oxygen consumption and lipid-rich content of the brain, the lack of an adequate antioxidant barrier makes the brain highly vulnerable to oxidative stress [12]. Oxidative stress is related to the physiological process of epilepsy [13], including focal epilepsy and generalized epilepsy. The oxidation–reduction homeostasis is maintained by both oxides and antioxidants. In mitochondria, ROS are produced by reduction of oxygen. There are a variety of defense mechanisms against ROS, including enzymatic and non-enzymatic antioxidants [14, 15]. The enzymatic antioxidants include SOD, glutathione transferase (GST), catalase (CAT) and the thioredoxin system. The non-enzymatic antioxidants include major antioxidants present in extracellular fluid, such as uric acid (UA), total bilirubin (TBIL), albumin (ALB), vitamins (e.g., vitamin A, vitamin C), and some metal ions including zinc. Previous studies have shown antioxidant abnormalities in patients with epilepsy. Specifically, compared with healthy individuals, patients with epilepsy have increased levels of TBIL [16] and UA [17], and significantly decreased levels of zinc and vitamin C [18]. Certain antioxidant genes are overexpressed in refractory epilepsy, such as GST [19]. ALB can resist oxidative stress [20, 21]. In addition, anti-epileptic treatment can lead to changes in oxidative status, such as decreases of serum zinc [22] and TBIL [23]. However, observational studies do not allow determination of a specific causal link between antioxidants and epilepsy, due to the inherent limitation of this research approach, which cannot exclude confounding factors. As a result, whether oxidative stress is a cause or a downstream effector of epilepsy remains unclear.

Mendelian randomization (MR) is a method to analyze causal effects between exposure and outcome, which uses genetic variants in genome-wide association studies (GWAS) as instrumental variables (IVs). In contrast to traditional epidemiological methods, the MR method can overcome potential confounding factors and reverse causality problems and is widely used for investigating causal effects. Previous studies have investigated the causal relationship between gut microbiota and epilepsy using the MR method and identified specific gut microbiota associated with an increased risk of epilepsy [24]. MR has also been employed to establish causal relationships between biomarkers and neurodegenerative diseases such as Parkinson's disease [25, 26], Alzheimer's disease [27], and ischemic stroke [28]. In this study, we performed a two-sample bidirectional MR analysis to explore the causal relationship between oxidative stress and epilepsy.

## Methods

### Study design

**Fig.1:**
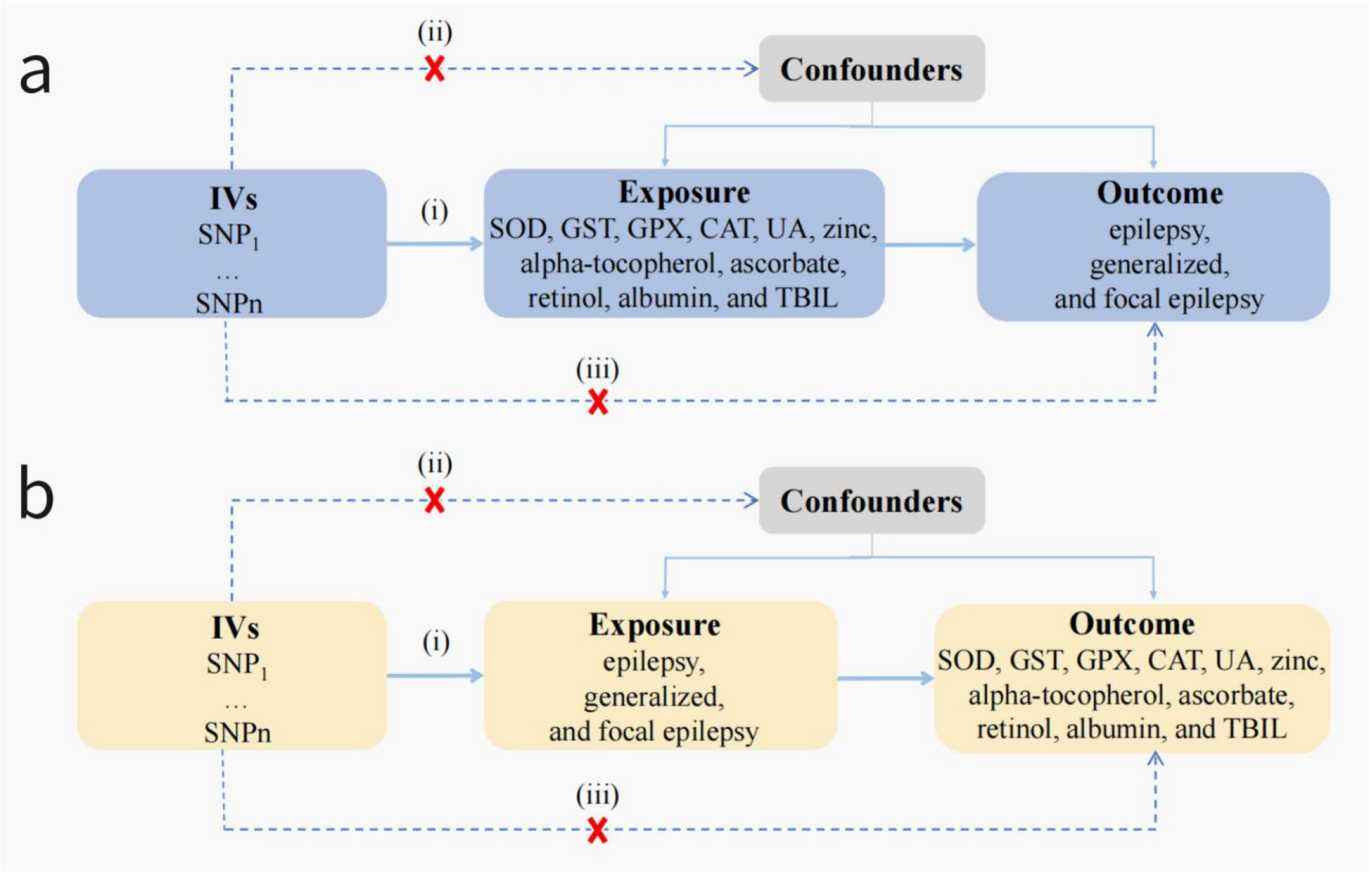
The framework of the bidirectional two-sample Mendelian randomization (MR) analysis between oxidative stress injury biomarkers and epilepsy. Three major assumptions of the MR analysis are as follows: (i): Genetic variation as an instrumental variable must be genuinely associated with exposures (oxidative stress injury biomarkers or epilepsy). (ii) Exposure-outcome confounders have no effect on genetic variation. (iii): Genetic variation affects outcomes through exposure only, independent of other pathways. IVs: Instrumental variables, SNPs: Single nucleotide polymorphisms, SOD: Superoxide dismutase, GST: Glutathione transferase, GPX: Glutathione per-oxidase, CAT: Catalase, UA: Uric acid, TBIL: Total bilirubin

In this MR study, IVs were selected based on three fundamental assumptions: (1) IVs are strongly associated with exposures; (2) IVs are not associated with confounders; and (3) IVs influence the outcome only through exposures. An overview of this study is presented in Fig. 1. All participants provided written informed consent in the corresponding original GWAS.

### GWAS statistics for oxidative stress biomarkers

GWAS data of 11 oxidative stress biomarkers were obtained from the open database (https://gwas.mrcieu.ac.uk/) according to a previous paper [15]. The 11 oxidative stress biomarkers were SOD, GST, glutathione peroxidase (GPX), CAT, UA, zinc, alpha-tocopherol, ascorbate, retinol, ALB, and TBIL.

### GWAS statistics for epilepsy and epileptic subtypes

The GWAS statistics for epilepsy (15,212 cases and 29,677 controls) and epileptic subtypes including generalized epilepsy (3769 cases and 29,677 controls) and focal epilepsy (9671 cases and 29,677 controls) were obtained from the International League Against Epilepsy consortium [29]. Most of the participants were of European ancestry. Detailed characteristics are shown in Table 1.

**Table 1:** Characteristics of studies and datasets used in this Mendelian randomization study

Exposure or Outcome	Population	Sample size	PMID	Source
**Oxidative stress**				
** biomarkers**				
SOD	European	3301	29,875,488	https://gwas.mrcieu.ac.uk/datasets/prot-a-2800/
GST	European	3301	29,875,488	https://gwas.mrcieu.ac.uk/datasets/prot-a-1283/
GPX	European	3301	29,875,488	https://gwas.mrcieu.ac.uk/datasets/prot-a-1265/
CAT	European	3301	29,875,488	https://gwas.mrcieu.ac.uk/datasets/prot-a-367/
UA	European	343,836	/	https://gwas.mrcieu.ac.uk/datasets/ukb-d-30880_raw/
Zinc	European	2630	23,720,494	https://gwas.mrcieu.ac.uk/datasets/ieu-a-1079/
Tocopherol	European	6226	24,816,252	https://gwas.mrcieu.ac.uk/datasets/met-a-571/
Ascorbate	European	64,979	/	https://gwas.mrcieu.ac.uk/datasets/ukb-b-19390/
Retinol	European	62,991	/	https://gwas.mrcieu.ac.uk/datasets/ukb-b-17406/
Albumin	European	115,060	/	https://gwas.mrcieu.ac.uk/datasets/met-d-Albumin/
TBIL	European	342,829	/	https://gwas.mrcieu.ac.uk/datasets/ukb-d-30840_raw/
**Epilepsy**				
Epilepsy (All documented cases)	Mixed	44,889	30,531,953	https://gwas.mrcieu.ac.uk/datasets/ieu-b-8/
Generalized epilepsy (All documented cases)	Mixed	33,446	30,531,953	https://gwas.mrcieu.ac.uk/datasets/ieu-b-9/
Focal epilepsy (All documented cases)	Mixed	39,348	30,531,953	https://gwas.mrcieu.ac.uk/datasets/ieu-b-10/

*SOD* Superoxide dismutase, *GST* Glutathione S-transferase, *GPX* Glutathione peroxidase, *CAT* Catalase, *UA* Uric acid, *TBIL* Total bilirubin

### MR design

A bidirectional MR was used to test the associations of oxidative stress biomarkers with epilepsy and epileptic subtypes, and vice versa. Single nucleotide polymorphisms (SNPs) related to exposures were selected as IVs based on three fundamental assumptions as shown above. We set *P* < 5 × 10^−8^ as the genome-wide significance threshold for UA and TBIL, and *P* < 5 × 10^−6^ for others to obtain enough SNPs strongly associated with oxidative stress. Meanwhile, independent SNPs related to exposures were gained after removing SNPs with linkage disequilibrium (*r* ^2^ < 0.001 and 1 Mb distance).* F*-statistic (β^2^/se^2^) was used to evaluate the strength of the association, and SNPs were deleted if their* F*-statistic was < 10 (Supplementary Tables S1–S6). Additionally, we scanned the PhenoScanner database to detect the associations between SNPs and other potential confounders.

### Statistical analyses

Inverse-variance weighted (IVW) method was used as a dominant analysis method to evaluate the causal association between oxidative stress biomarkers and epilepsy. MR-Egger, weighted-median method, simple mode method and weighted mode method were used as complementary methods. The Cochrane’s Q test was performed to evaluate the heterogeneity of the MR assumptions. The MR-Egger intercept test was conducted to detect horizontal pleiotropy. The Mendelian Randomization Pleiotropy RESidual Sum and Outlier (MR-PRESSO) examination was performed to assess the total pleiotropy to ensure a robust estimate. Moreover, “leave-one-out” analyses were conducted to evaluate the stability of the MR assumptions. All the above statistical analyses were conducted using the “Two Sample MR” and “MR-PRESSO” packages in R software (version 4.3.1). Since the risk of epilepsy was dichotomous variables, we used the odds ratios (ORs) and 95% confidence intervals (CIs) to present the causal effects of 11 oxidative stress biomarkers on the risk of epilepsy. The Bonferroni method was used for multiple comparisons, and a *P* value less than 0.00076 (0.05/66) was considered as statistically significant evidence of a causal association. A* P* value of less than 0.05 was considered as suggestive for a potential causal association.

## Results

### Causal effects of oxidative stress biomarkers on epilepsy and its subtypes

**Fig. 2:**
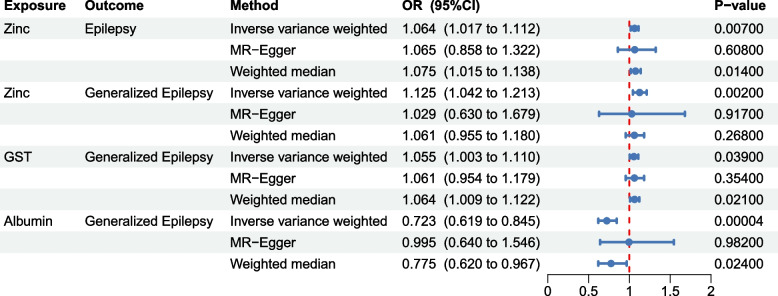
Mendelian randomization results of the associations of oxidative stress injury biomarkers with the risk of epilepsy. GST: Glutathione S-transferase

The results of MR analysis are summarized in Fig. 2 and Supplementary Table S1. The IVW method showed that ALB was significantly associated with a decreased risk of generalized epilepsy (OR = 0.723, 95% CI 0.619–0.845, *P* = 4.26E-05). In addition, zinc was potentially associated with increased risks of epilepsy and the generalized epilepsy subtype (OR = 1.064, 95% CI 1.017–1.112, *P* = 0.007; OR = 1.125, 95%CI 1.042–1.213,* P* = 0.002). GST was potentially associated with an increased risk of generalized epilepsy subtype (OR = 1.055, 95%CI 1.003–1.11, *P* = 0.039). There was no causal effect of oxidative stress on focal epilepsy (*P* > 0.05).

A series of sensitivity analyses including heterogeneity and pleiotropy analyses were conducted to evaluate any bias of the MR assumptions. The Cochrane’s Q test suggested the existence of heterogeneity (including UA on epilepsy and its subtypes, retinol on epilepsy and its subtypes, ALB on epilepsy, and GPX on generalized epilepsy), so we conducted the study using the random-effects IVW method. The MR-Egger intercept suggested no horizontal pleiotropy in our study. The MR-PRESSO test found no outliers, and the global test showed no pleiotropy. The detailed results of sensitivity analyses are shown in Supplementary Table S1. Besides, the scatter and forest plots of MR analysis are presented in Figs. S1 and S2. The funnel plots demonstrated no evidence of asymmetry, indicating a low risk of directional pleiotropy (Fig. S3). The leave-one-out analysis did not detect significant outliers (Fig. S4).

### Reversed two-sample MR analysis

**Fig. 3:**
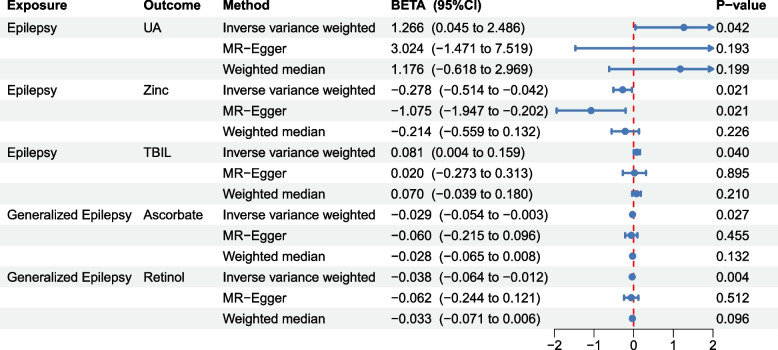
Mendelian randomization results of the association of epilepsy with various oxidative stress injury biomarkers. UA: uric acid, TBIL: total bilirubin

In the MR analysis from epilepsy and its subtypes to oxidative stress, no significant association was found based on the Bonferroni-corrected threshold (Fig. 3); however, there was a potential causal effect of epilepsy on UA and TBIL. Epilepsy was potentially associated with increased UA and TBIL levels (beta = 1.266, 95% CI 0.045 – 2.486, *P* = 0.042; beta = 0.081; 95% CI 0.004 – 0.159, *P* = 0.04). The results also showed a potential causal relationship between epilepsy and zinc level (beta =  − 0.278, 95% CI − 0.514 to − 0.042, *P* = 0.021). The generalized epilepsy was potentially associated with decreased ascorbate and retinol levels (beta =  − 0.029, 95% CI − 0.054 to − 0.003, *P* = 0.027; beta =  − 0.038; 95% CI ranges from − 0.064 to − 0.012, *P* = 0.004). There was no evidence of the effect of focal epilepsy on oxidative stress biomarkers (*P* > 0.05) in Supplementary Table S1. The Cochrane’s Q test indicated no heterogeneity. There was no evidence of horizontal pleiotropy in the MR-Egger intercept test (Supplementary Table S1). In addition, except for the SNP rs61779328 of epilepsy, no outliers were found by the MR-PRESSO test.

## Discussion

Although a multitude of observational studies have explored associations between oxidative stress and epilepsy, few relevant MR studies have been conducted. Some MR studies focused on the causal relationship of serum metabolites or circulating lipids with epilepsy. However, these studies employed a unidirectional approach [30]. In this study, we performed for the first time bidirectional MR analyses to systematically evaluate the causal effect of 11 oxidative stress injury biomarkers on epilepsy and epileptic subtypes, including generalized epilepsy and focal epilepsy. By using SNPs as IVs, we found that serum zinc is potentially associated with an increased risk of both generalized and focal epilepsy. GST is associated with an increased risk of generalized epilepsy, while ALB is associated with a decreased risk of generalized epilepsy. The potential causal effects of epilepsy on oxidative stress biomarkers were also evaluated, showing that epilepsy is potentially associated with increased UA and TBIL as well as decreased zinc. In addition, generalized epilepsy is potentially associated with decreased levels of both vitamin C and vitamin A. These findings point to the imbalance between the oxidative damage caused by peroxides and the oxidative defense as the potential cause of epilepsy. We found that oxidative stress plays an important role in epilepsy, and previous studies have also shown that antioxidant therapy can improve epilepsy [31].

The antioxidant system of mitochondria can suppress excess ROS, but this capacity is rather limited. Hence, antioxidants are needed to counteract oxidative damage.

GSTs are enzymatic antioxidants that catalyze the conjugation of non-polar compounds that containing an electrophilic carbon, nitrogen or sulfur atom to reduced GSH. When exposed to a pro-oxidative environment, increased production of GSTs occurs to resist oxidative stress [32]. Our results showed that the GST level was positively associated with an increased risk of generalized epilepsy. A previous study has found up-regulation of GST expression in a *Drosophila* model of drug-resistant epilepsy [19]. In a rat model of trauma, GST content in the hippocampus showed time-dependent changes as early as 3 h post-trauma, accompanied by a synchronous increase in oxidative substances, and reached a peak at 24 – 48 h [33]. Therefore, we speculate that the body of patients with higher levels of GST is involved in more oxidative stress processes, and they produce more ROS, thereby increasing the risk of epilepsy. Overall, interventions targeting the GST level may affect progression of epilepsy.

The second line of defense, non-enzymatic antioxidants such as ALB, zinc, UA, TBIL, retinol, and ascorbate, play important roles in the antioxidant system. They can quickly inactivate free radicals and resist oxidants [34].

ALB is the main thiol antioxidant in humans, which offers resistance to cell damage by hydrogen peroxide and rebalances the redox state in the body [20]. Astrocytes can promote seizures and inflammatory responses in mice with temporal lobe epilepsy by activating the NF-κB signaling pathway [35], while ALB can reduce ROS by inhibiting the activation of NF-κB pathway [36]. Consistently, here we found that patients with elevated ALB levels had a reduced risk of seizures. By using the MR method, we provided more definitive evidence for the association between ALB and epilepsy. Some antioxidant compounds have been tested as potential countermeasures for central nervous system diseases. In addition, many clinical trials have shown that exogenous supplementation of antioxidants by the nano-carrier technology can ameliorate central nervous system diseases [37]. Therefore, supplementation of ALB may be a potential treatment strategy for epilepsy.

Zinc is essential for normal brain development and plays a key role in oxidative stress regulation and neuronal signaling. Zinc has also been found to impact not only the occurrence of epilepsy but also the comorbidity of epilepsy with depressive disorders and cognitive impairment [38]. Abnormal zinc levels may lead to neuronal dysfunction or degeneration, impairing the regulation of epileptic-related ligands and voltage-gated channels [39]. Despite the wealth of data on the effects of zinc on epilepsy, the results are somewhat controversial. Kasaragod et al. found that zinc inhibits gamma-aminobutyric acid (GABA) receptor function by impeding chloride conductance and weakening inhibitory nerve impulse conduction [40]. In brain slices from a rat model of epilepsy, zinc induces collapse of augmented inhibition by GABA, indicating its involvement in the promotion of seizures [41]. In another study of three different rat epilepsy models induced by auditory stimulation or electric shock [42], the plasma levels of zinc were significantly lower than that of the control group, suggesting reduced zinc concentrations after seizures. Zinc is involved in a variety of mechanisms affecting epilepsy, such as regulation of *N*-methyl-*D*-aspartic acid (NMDA) receptors and GABA receptors. The presence of these multiple mechanisms makes it challenging to precisely measure the net effect of zinc and to understand its complex relationship with epilepsy [38]. More clinical studies are needed to explore the changes of serum zinc before and after seizures. In our MR study without residual confounding or reverse causality, we found that elevated zinc levels promoted both generalized and focal seizures, whereas serum zinc level decreased after an episode. More clinical research is needed to explore the changes in serum zinc before and after seizures.

During status epilepticus or generalized tonic-clonic seizures, the serum level of UA increases significantly, which may even lead to acute renal failure due to hyperuricemia [17]. This is also confirmed by our experimental results. UA is not only an antioxidant [43], but also the end product of purine metabolism. Tissue hypoxia caused by epileptic seizures accelerates the breakdown of purines in the liver, which results in hyperuricemia in epilepsy patients [44]. Therefore, the presence and severity of limb tics in patients with impaired consciousness can also be determined by a rapid test of serum levels of UA in the absence of family members observing their seizures.

Our study found a positive association between epilepsy and elevated TBIL. TBIL is considered to be toxic to the brain, but its antioxidant effect cannot be ignored. In fact, bilirubin contains reactive hydrogen atoms and conjugated double bond systems, and it has been observed that bilirubin can effectively capture peroxygenic radicals in the lipid peroxidation system [45]. TBIL can reduce oxidative stress and inhibit the inflammatory state. Metabolic studies have found that compared with healthy controls, patients with epilepsy have increased oxidative stress and elevated bilirubin content in the blood [16]. After antiepileptic treatment, bilirubin decreases significantly [23]. At the same time, a large number of clinical observations have found that newborns with jaundice are more likely to have seizures after phototherapy [46], which may be caused by the low phototherapy threshold, significantly reduced bilirubin level, and weakened antioxidant capacity. Therefore, the increase of TBIL during epilepsy may be a response of the antioxidant system.

Retinol is also known as vitamin A. It regulates the expression of a variety of genes, including those involved in the oxidative stress response. Thus, vitamin A is an indirect antioxidant that transcriptionally regulates a number of genes involved in the antioxidant response [47]. Epilepsy is a nervous system disease with strong involvement of oxidative stress [13, 48], which may increase the content of ROS and the production of super-oxides in the brain. The decrease in plasma vitamins may be an important factor contributing to the increased sensitivity of the body to lipid per-oxidation.

Water-soluble vitamin C, also known as ascorbic acid (AA) [49], participates in important biological processes such as synthesis of neurotransmitters and paracrine lipid mediators, and epigenetic regulation of deoxyribonucleic acid (DNA). AA is neuroprotective as a prototypical antioxidant and is involved in redox-coupled reactions. Studies in animal models have shown that AA has potent anticonvulsant properties. High doses of AA have been suggested as a possible supplementary therapy for epilepsy. The anticonvulsant effects of AA may be mediated through the modulation of glutamate release in the hippocampus by the glutamate-AA hetero-exchange system [48]. Ascorbate reduces the levels of free radicals produced by glutamate, inhibits the binding of glutamate to synaptic receptors, modifies the activity of glutamate receptors, reduces stimulation of NMDA receptors, and protects neurons from glutamate excitotoxicity [50]. It is well-known that activation of glutamate receptors and excitotoxicity contribute to oxidative stress in experimental epileptic models, leading to lipid peroxidation and oxidative DNA damage [51, 52]. The generation of reactive ROS after a seizure attack plays a significant role in neuronal cell death [52]. AA is essential for preventing oxidative brain damage during seizures and maintaining the neuronal redox equilibrium. Few studies have found a direct association between epilepsy and vitamins C and A. The present MR study, which excluded confounding factors, revealed a causal relationship between increased susceptibility to epilepsy and decreased vitamin C and A levels.

A study suggests that drug-targeted oxidative stress improves seizure frequency in epileptic rat models [21]. After excluding reverse causality and confounding factors, our data suggest a relationship between endogenous antioxidants and epilepsy. Therefore, early treatment with antioxidant-targeting therapies may improve the long-term prognosis of epilepsy [53]. In addition, we can treat refractory epilepsy by ameliorating oxidative stress, thereby improving the patients' quality of life.

There are several advantages of our study. First, in this MR Study, we covered 11 oxidative stress biomarkers and two epilepsy subtypes, making it the most comprehensive MR study of the relationship between oxidative stress and epilepsy. Second, studies using MR designs can avoid reverse causality problems and residual confounding. In addition, there are also some limitations. First, the effect of oxidative stress varies dynamically in individuals in this study. Second, the population included in this study is largely European. The results of MR should be further validated in other populations to increase the generality of GWAS data. Finally, we did not find a causal association of SOD, GPX, CAT or alpha-tocopherol with epilepsy. More accurate laboratory studies are needed to determine the biochemical basis of these associations.

## Conclusions

In conclusion, in this bidirectional MR study, we showed a significant causal relationship between ALB and generalized epilepsy. Zinc was potentially associated with increased risks of epilepsy and generalized epilepsy. GST was potentially associated with increased risk of generalized epilepsy. Reverse MR analysis showed some potential causal effects of epilepsy and its subtypes on oxidative stress biomarkers. Our results suggest that seizures can lead to elevated uric acid levels. GST may be a therapeutic target to affect the progression of epilepsy. ALB antioxidant may be an add-on therapy for epilepsy treatment. This study also provides reliable evidence for the relationship between TBIL and epilepsy, and adds to the evidence of the causal relationship of vitamin C and vitamin A with epilepsy.

## Supplementary Information

Supplementary Table S1.

Supplementary Figure S1.

Supplementary Figure S2.

Supplementary Figure S3.

Supplementary Figure S4.

## Data Availability

All data used in this study are publicly available, with relevant citations detailed.

## Abbreviations

AA: Ascorbic acid
ALB: Albumin
CAT: Catalase
CI: Confidence intervals
DNA: Deoxyribonucleic acid
GABA: Gamma-aminobutyric acid
GLUT1: Glucose transporter type 1
GWAS: Genome-wide association studies
GPX: Glutathione peroxidase
GSH: Glutathione
GST: Glutathione transferase
IVs: Instrumental variables
IVW: Inverse-variance weighted
MR: Mendelian randomization
MR-PRESSO: Mendelian Randomization Pleiotropy RESidual Sum and Outlier
NMDA: N-methyl-D-aspartic acid
OR: Odds ratio
ROS: Reactive oxygen species
SNPs: Single nucleotide polymorphisms
SOD: Superoxide dismutase
TBIL: Total bilirubin
UA: Uric acid
