# Supplementary material for: The associations between oxidative stress and epilepsy: a bidirectional two-sample Mendelian randomization study
Source: Acta Epileptol. 2024 Dec 1;6:33. doi: 10.1186/s42494-024-00173-4 (PMC11960306; doi:10.1186/s42494-024-00173-4)
Supplement: Supplementary file 3 — Supplementary Figure S2. [file 42494_2024_173_MOESM3_ESM.docx]

Supplementary Figure 2


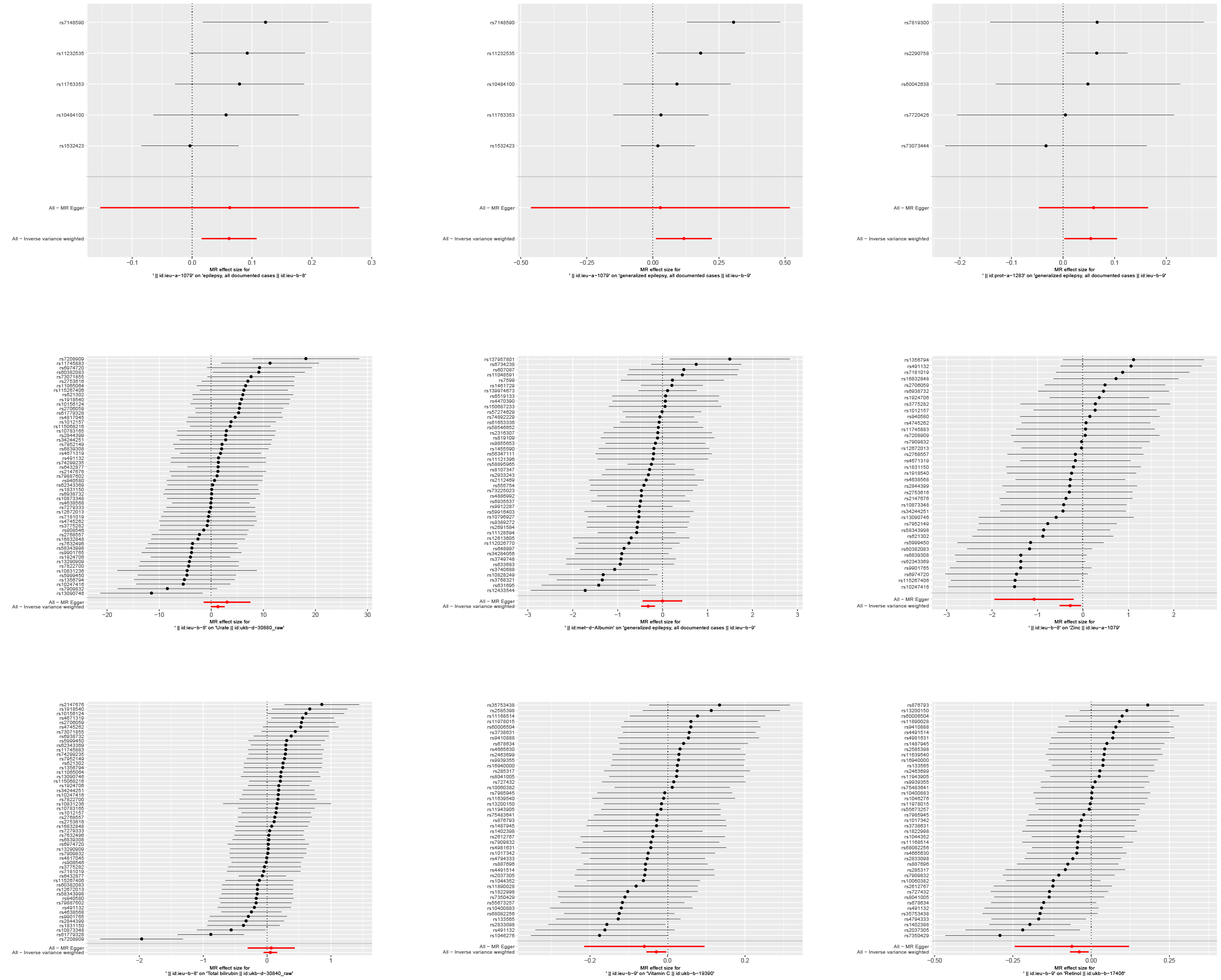


Figure S2: Forest plots of MR analysis. Top (from left to right), forest plots of the associations of zinc with epilepsy and generalized epilepsy, and GST with generalized epilepsy. Middle (from left to right), scatter plots of albumin on generalized epilepsy, epilepsy on urate and epilepsy on zinc. Bottom (from left to right), scatter plots of epilepsy on TBIL, generalized epilepsy on ascorbate, and generalized epilepsy on retinol. MR, mendelian randomization; GST, glutathione transferase; TBIL, total bilirubin.
