# Supplementary material for: The associations between oxidative stress and epilepsy: a bidirectional two-sample Mendelian randomization study
Source: Acta Epileptol. 2024 Dec 1;6:33. doi: 10.1186/s42494-024-00173-4 (PMC11960306; doi:10.1186/s42494-024-00173-4)
Supplement: Supplementary file 4 — Supplementary Figure S3. [file 42494_2024_173_MOESM4_ESM.docx]

Supplementary Figure 3


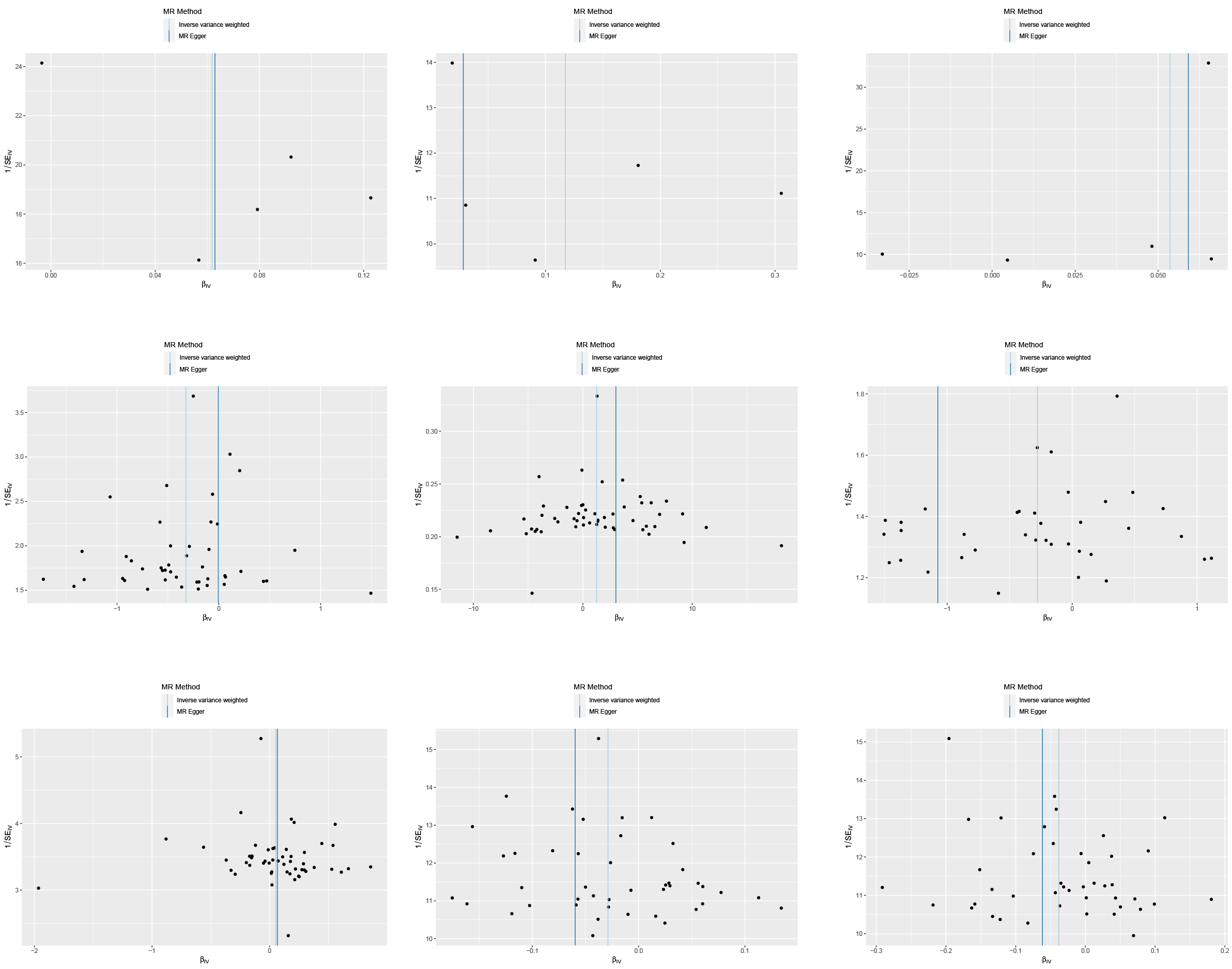


Figure S3: Funnel plots of MR analysis. Top (from left to right), funnel plots of the associations of zinc with epilepsy and generalized epilepsy, and GST with generalized epilepsy. Middle (from left to right), scatter plots of albumin on generalized epilepsy, epilepsy on urate and epilepsy on zinc. Bottom (from left to right), scatter plots of epilepsy on TBIL, generalized epilepsy on ascorbate, and generalized epilepsy on retinol. MR, mendelian randomization; TBIL, total bilirubin; GST, glutathione transferase.
