# Supplementary material for: The associations between oxidative stress and epilepsy: a bidirectional two-sample Mendelian randomization study
Source: Acta Epileptol. 2024 Dec 1;6:33. doi: 10.1186/s42494-024-00173-4 (PMC11960306; doi:10.1186/s42494-024-00173-4)
Supplement: Supplementary file 5 — Supplementary Figure S4. [file 42494_2024_173_MOESM5_ESM.docx]

Supplementary Figure 4


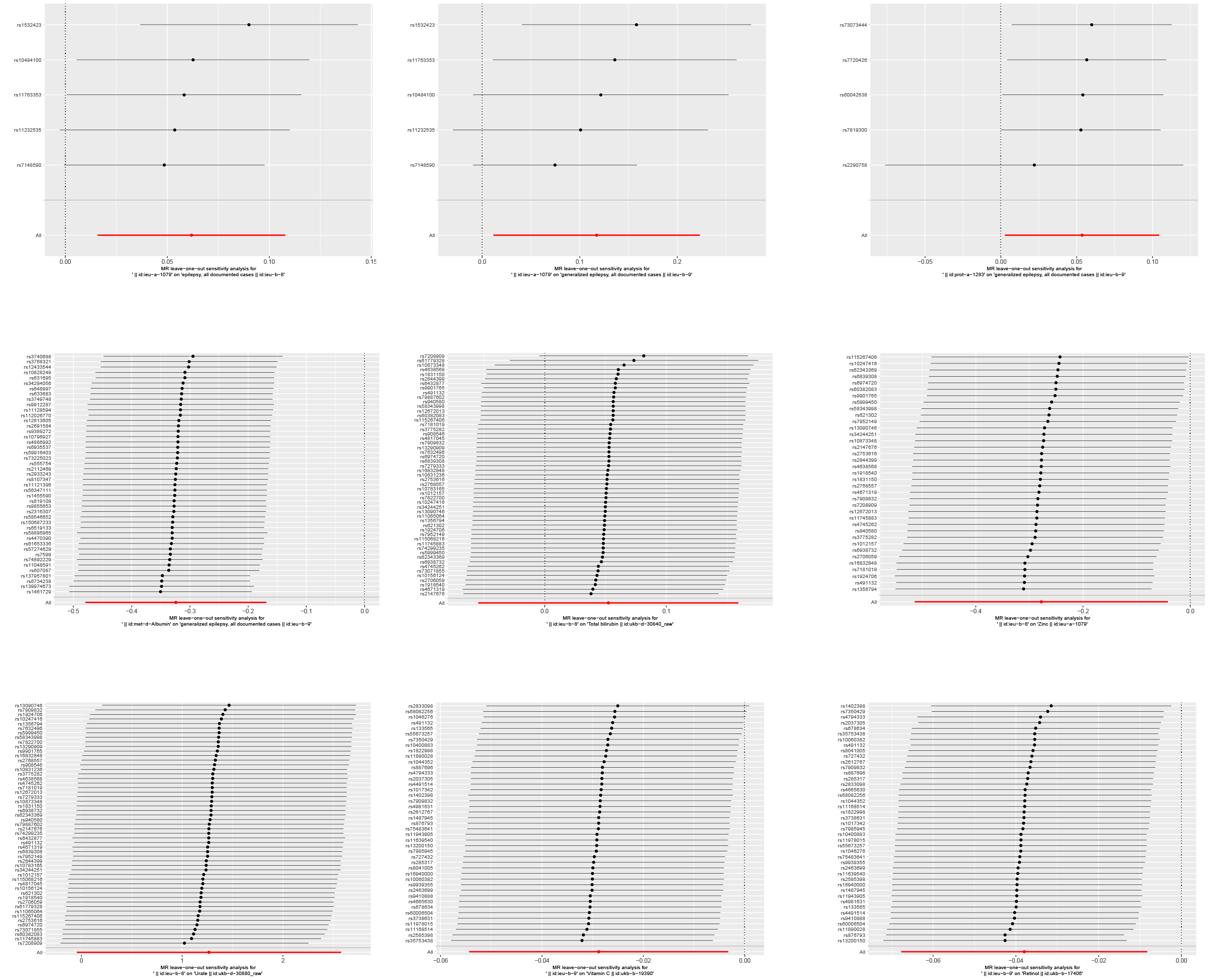


Figure S4: MR leave-one-out sensitivity analysis. Top (from left to right), leave-one-out sensitivity analysis of zinc on epilepsy, zinc on generalized epilepsy and GST on generalized epilepsy. Middle (from left to right), scatter plots of albumin on generalized epilepsy, epilepsy on urate and epilepsy on zinc. Bottom (from left to right), scatter plots of epilepsy on TBIL, generalized epilepsy on ascorbate, and generalized epilepsy on retinol. MR, mendelian randomization; TBIL, total bilirubin; GST, glutathione transferase.
